# Prognostic Significance of Tumor-Associated Macrophage Content in Head and Neck Squamous Cell Carcinoma: A Meta-Analysis

**DOI:** 10.3389/fonc.2019.00656

**Published:** 2019-07-23

**Authors:** Ayan Tyagi Kumar, Alexander Knops, Brian Swendseid, Ubaldo Martinez-Outschoom, Larry Harshyne, Nancy Philp, Ulrich Rodeck, Adam Luginbuhl, David Cognetti, Jennifer Johnson, Joseph Curry

**Affiliations:** ^1^Department of Otolaryngology-Head and Neck Surgery, Thomas Jefferson University, Philadelphia, PA, United States; ^2^Sidney Kimmel Medical College, Thomas Jefferson University, Philadelphia, PA, United States; ^3^Department of Medical Oncology, Thomas Jefferson University, Philadelphia, PA, United States; ^4^Department of Neurological Surgery, Thomas Jefferson University, Philadelphia, PA, United States; ^5^Department of Pathology, Anatomy, and Cell Biology, Thomas Jefferson University, Philadelphia, PA, United States; ^6^Department of Dermatology, Thomas Jefferson University, Philadelphia, PA, United States

**Keywords:** tumor microenviroment, tumor associated macrophage (TAM), head and neck (H&N) cancer, CD68, CD163, M1 macrolphage, M2 macrophage

## Abstract

**Background:** Head and neck squamous cell carcinoma (HNSCC) exists within a microenvironment rich in immune cells. Macrophages are particularly abundant in and around tumor tissue, and have been implicated in the growth, malignancy, and persistence of HNSCC (1). However, current literature reports variable degrees of association between the density of tumor-associated macrophages (TAMs) and clinicopathologic markers of disease (2, 3). These inconsistent findings may be a result of differences in approach to TAM detection. Authors have measured total TAMs in tumor tissue, while others have stained tumor samples for individual subtypes of TAMs, which include pro-inflammatory (M1-like) and immunosuppressive (M2-like). Our aim is to more clearly define the prognostic significance of the phenotypes of tumor-associated macrophages in HNSCC.

**Methods:** We conducted a meta-analysis of the existing publications investigating the relationship between TAMs (total and M2-like subtype) and T stage, nodal involvement, vascular invasion, lymphatic invasion, and tumor differentiation (**Figure 1**). A total of 12 studies were included. Forest plots and risk ratios were generated to report overall effect.

**Results:** Higher density of both total and M2-like subtype of TAMs in the tumor microenvironment is associated with advanced T stage, increased rates of nodal positivity, presence of vascular invasion, and presence of lymphatic invasion (*p* < 0.0001; **Figures 2–9**). There is no significant association between TAM density, either total or M2-like subtype, and tumor differentiation (**Figures 10**, **11**).

**Conclusions:** Increased density of TAMs, including those of the M2-like phenotype, correlate with poor clinicopathologic markers in HNSCC. Our findings warrant additional investigation into the subpopulations of TAMs, the mechanisms behind their recruitment and differentiation, and the associated influence of each phenotype on tumor growth and invasion. A greater understanding of TAM dynamics in HNSCC is critical for directing further research and employing TAM-targeted adjunct therapies.

## Background

The tumor microenvironment (TME) is comprised of various cellular components with complex interactions between these components and tumor cells. In 1889, Paget first described the “seed and soil” hypothesis, wherein carcinomas induce changes in adjacent stromal and inflammatory cells, which contribute to neoplastic growth and invasion (1–3). Tumor associated macrophages (TAMs) are one such critical component of the TME. TAMs are macrophages present in close proximity to tumor cells which play important roles in influencing host immune response to cancer. Macrophages, like many other immune effector cells, exist as multiple subtypes with differing expression patterns, surface markers, and secretable factors. The role they play in the TME depends on the phenotype of the macrophage. They are broadly categorized into two types, though this is a matter of debate and many subtypes exist. The first type is “classically activated,” or M1-like macrophages, and these stand in contrast to “alternatively activated,” or M2-like macrophages. M1-like macrophages are pro-inflammatory and are thought to exert antitumor effects through production of IL-12, IL-23, IFN-γ, and reactive oxygen and nitrogen species (4). Studies in multiple cancer types, including non-small cell lung, ovarian, and colorectal cancers, have correlated extended survival with presence of predominantly M1-like TAMs in the TME (5–7) Cumulatively, “classical” M1-like macrophages elicit tumor tissue disruption and may be considered host protective (8). M2-like macrophages inhibit M1-like TAMs and promote tissue remodeling (4, 9) through production of IL-10, TGF-β, VEGF, and TNF-α, and induction of angiogenesis (9, 10). In many tumors, infiltrating macrophages are predominantly of the “alternative” M2-like type, providing an immunosuppressive environment suitable for tumor growth (11). All TAMs, including both the M1-like and M2-like subtypes, can be identified and quantified by CD68 immunostaining, whereas M2-like macrophages are additionally and specifically characterized by CD163 surface marker (12, 13). Although other surface markers may be utilized to detect these subpopulations of macrophages, CD163 and CD68 are the most commonly employed for M2-like and total TAM identification, respectively. When present in high numbers, TAMs are associated with poor survival outcomes and the promotion of metastasis, angiogenesis, and invasion into nearby tissues and vasculature across many cancer types (2, 14, 15).

In head and neck squamous cell carcinoma (HNSCC), TAMs are recruited to the tumor microenvironment and directly contact SCC cells. These cells have been shown to promote disease progression and relapse, cellular dedifferentiation, and angiogenesis in HNSCC (16–18). Tumors demonstrating high levels of CD68 and CD163 immunostaining, representing total TAM and M2-like macrophage populations respectively, correlate with increased lymph node metastasis, extracapsular extension, and advanced stage (19). Poor cellular differentiation, advanced T and N stage, lymphovascular invasion are predictive of shorter survival in patients with HNSCC (20–25). Presence of TAMs in high numbers in the tumor microenvironment may thus be viewed as an indicator of poor prognosis (26). Current data suggest that CD163-positive protumor macrophages dominate the population of tumor associated macrophages (27, 28). Increased levels of these M2-like macrophages have been associated with high pathological grade, tumoral angiogenesis, recurrence after radiotherapy, poor response to chemotherapy, and decreased overall survival (26, 29–32). However, TAMs exist on a dynamic continuum between the two phenotypes, M1-like and M2-like, and their differentiation is not fixed (6, 8, 9). Studies have shown that specific signals can shift M2-like macrophage populations toward the M1-like phenotype, or even inhibit the polarization to M2-like subtype entirely (33, 34). Therefore, TAMs may represent a therapeutic target for various types of cancers, including HNSCC. There are multiple stages during which TAMs may be targeted for treatment, including recruitment to target tissue via CCL2/CCL8-CCR2 pathways, prevention of differentiation to the M2-like type, and direct induction of phenotypic M2-to-M1 reprogramming, possibly through Wnt/Beta-catenin pathway inhibition (34, 35). Clinical assessment of specific agents targeting TAMs is currently underway (36).

The aim of this meta-analysis is to define the prognostic significance of TAM populations in HNSCC by compiling existing data on the effect of total TAM (CD68+) and M2-like TAM (CD163+) density on burden of disease and pathologic markers of tumor aggressiveness.

## Methods

**Figure 1:**
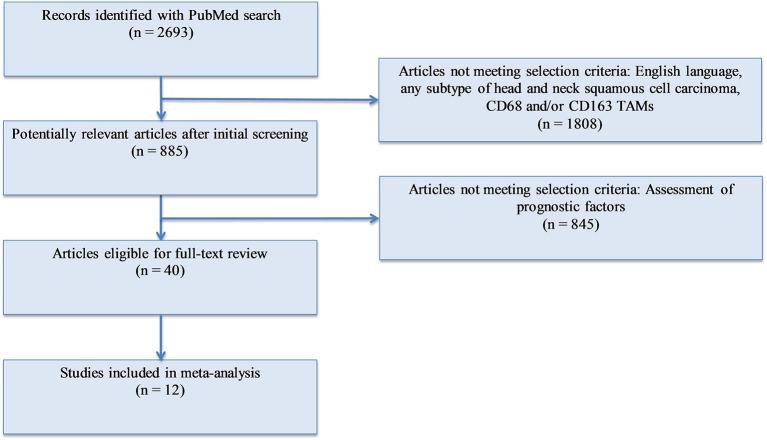
Flowchart of systematic review.

A PubMed search was conducted with the following keywords: (“Tumor associated macrophages” OR “TAM” OR “M2” OR “CD163” OR “CD68”) AND (“squamous cell carcinoma”). PRISMA recommendations were followed (37). Of the articles found, studies were selected for analysis using the following criteria: (1) English language; (2) human subjects; (3) squamous cell carcinoma of the head and neck; (4) measurement of either CD68+ or CD163+ tumor associated macrophages or both; (5) available data on at least one of the following pathological markers: T stage, N stage, differentiation, lymphatic vessel invasion, vascular invasion. After identification, screening, and evaluation of eligibility, 12 studies were selected for meta-analysis (Figure 1). All immunohistochemistry was performed on tumor samples from patients with HNSCC. A total of 1,551 patients were assessed, with 945 having oral SCC, 500 with esophageal SCC, and 106 with unspecified HNSCC. CD68 was utilized as the panmacrophage marker, including M1-like and M2-like subtypes, and CD163 represented only the M2-like population of TAMs in all studies. CD68 and CD163 detection was reported by pathologist scoring or software calculation. Seven studies utilized median values of marker density while four studies utilized mean values of marker density to distinguish high vs. low CD68 or CD163 staining. One study (38) utilized an online software-based method established by Budczies et al. (39) to distinguish high vs. low density. Based on classifications outlined by the selected studies, high T stage was defined as T3 or T4, nodal status was grouped as positive or negative, lymphatic invasion and vascular invasion were deemed present or not present, and tumors were pathologically classified as poorly or well differentiated. All data was entered into Review Manager 5.3 (Nordic Cochrane Centre, Copenhagen, Denmark) in order to construct Forest Plots. Forest Plot specifications were as follows: dichotomous for data type, fixed effect for analysis methods, and risk ratio for effect measure.

## Results

The characteristics of the 12 studies selected for meta-analysis are summarized in Table 1 (14, 38, 40–49). The articles constituted a total sample size of 1,551 patients. In all but three studies, patients had not received therapy prior to surgery. Four studies evaluated CD68 density; two studies evaluated CD163 density; six studies evaluated both markers in relation to clinicopathologic factors of HNSCC.

**Table 1:** Characteristics of studies evaluating TAM density and clinicopathologic markers in HNSCC.

**References**	**Number of patients**	**Site of cancer**	**Age of patients**	**TAM markers assessed**	**Prior therapy received**	**TAMs high-low distinction**
Balermpas et al. (40)	106	HNSCC	Not reported	CD68, CD163	Chemoradiotherapy	> or < than median value of all patients
Fang et al. (41)	78	Oral SCC	60 (24–82)	CD68	No neoadjuvant therapy	> or < than mean value of all patients
Fujii et al. (42)	108	Oral SCC	66.4 (23–93)	CD68, CD163	No neoadjuvant therapy	> or < than mean value of density
Hu et al. (43)	127	Oral SCC	61 (34–88)	CD68, CD163	No neoadjuvant therapy	> or < than mean value of density
Liu et al. (44)	112	Oral SCC	Not reported	CD68	No neoadjuvant therapy	> or < than median value of density
Lu et al. (14)	92	Oral SCC	51 (21–76)	CD68	No neoadjuvant therapy	> or < than median value of density
Matsuoka et al. (45)	60	Oral SCC	68.9 (33–87)	CD163	Chemoradiotherapy	> or < than median value of density
Shigeoka et al. (46)	70	Esophageal SCC	65.7 (54–88)	CD68, CD163	No neoadjuvant therapy	> or < than median value of density
Sugimura et al. (32)	210	Esophageal SCC	154 patients <70, 56 patients >70	CD68, CD163	104 received prior chemotherapy	> or < than median value of density
Wang et al. (47)	298	Oral SCC	53 (21–78)	CD163	No neoadjuvant therapy	> or < than mean value of density
Yamagata et al. (48)	70	Oral SCC	28–84	CD68, CD163	No neoadjuvant therapy	> or < than median value of density
Zhu et al. (38)	220	Esophageal SCC	124 patients <60, 96 patients >60	CD68	No neoadjuvant therapy	Method established by Budczies et al. (http://molpath.eharite.de/cutoff/)

### Increased CD68+ and CD163+ Density Is Associated With Advanced T Stage

**Figure 2:**
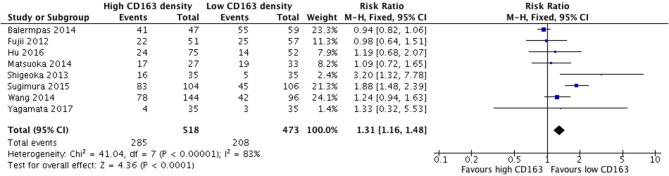
High CD68+ TAM density correlates with advanced T stage (T3, T4).

**Figure 3:**
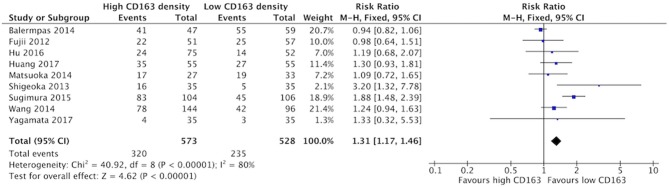
High CD163+ TAM density correlates with advanced T stage (T3, T4).

Increased presence of tumor-associated macrophages (CD68+) was associated with stage T3 and T4 HNSCC (Figure 2). Of the nine identified studies that compared high vs. low CD68+ immune cell density, eight suggested a greater risk of high T stage with increased TAMs. The combined risk ratio of these studies was 1.42 (95%CI = 1.25–1.62), representing a statistically significant correlation between high total TAM number and high T stage (*p* < 0.00001). The average rate of advanced T stage was 52.6% in those samples with high CD68 density, and 37.6% in samples with low CD68 density. Greater density of M2-like TAMs (CD163+) was associated with higher T stage (Figure 3). Eight studies compared the density of CD163+ and T stage with a cumulative risk ratio of 1.31 (95% CI = 1.16–1.48, *p* < 0.0001). The average rate of advanced T stage was 55.3% in those samples with high CD163 density, and 44% in samples with low CD163 density. Taken together, increased presence of TAMs, and specifically the M2-like subtype, is associated with larger and more locally invasive primary tumors.

### Increased CD68+ and CD163+ Density Is Associated With Nodal Metastasis

**Figure 4:**
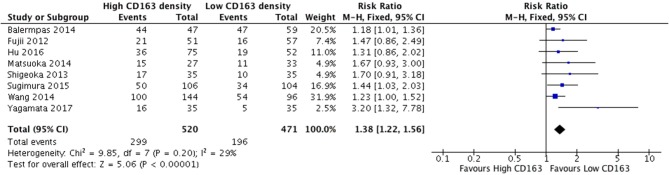
High CD68+ TAM density is associated with nodal positivity.

**Figure 5:**
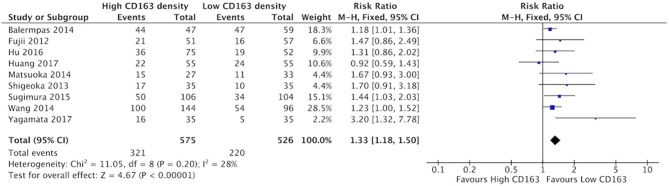
High CD163+ TAM density is associated with nodal positivity.

The presence of high numbers of CD68+ TAMs in primary tumor site was associated with nodal metastasis (Figure 4). Eight studies investigated the relationship of CD68+ and N stage, of which seven demonstrated correlation to nodal positivity. Cumulatively, high density of CD68+ TAMs was correlated with a risk ratio of 1.42 (95%CI = 1.23–1.65, *p* < 0.00001). The average rate of nodal positivity was 56.3% in samples with high CD68 density, and 36.8% in samples with low CD68 density. High CD163+ staining in primary tumor site was associated with positive nodal metastasis in all eight of the studies that examined this relationship (Figure 5). The combined risk ratio for all studies was 1.38 (95%CI = 1.22–1.56, *p* < 0.00001). The average rate of nodal positivity was 55% in samples with high CD163 density, and 34% in samples with low CD163 density. Overall, higher numbers of TAMs, and specifically M2-like polarized TAMs, correlates with higher rates of nodal metastasis in HNSCC.

### Increased CD68+ and CD163+ Density Is Associated With Higher Rate of Vascular Invasion

**Figure 6:**
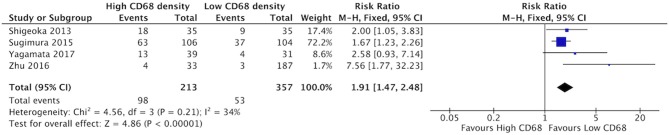
High CD68+ TAM density is associated with higher rates of vascular invasion.

**Figure 7:**
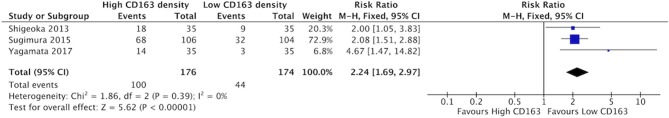
High CD163+ TAM density is associated with higher rates of vascular invasion.

High staining of CD68+ macrophages was associated with increased rate of vascular invasion in HNSCC (Figure 6). All four identified studies demonstrated risk ratios >1, with a cumulative ratio of 1.91 (95%CI = 1.47–2.48, (*p* < 0.00001). The average rate of vascular invasion was 39.1% in samples with high CD68 density, and 18.9% in samples with low CD68 density. Increased staining for CD163+ was associated with increased risk of vascular invasion (Figure 7). Three studies contributed to a risk ratio of 2.24 (95%CI = 1.69–2.97, *p* < 0.00001). The average rate of vascular invasion was 51.9% in samples with high CD163 density, and 21.7% in samples with low CD163 density. Cumulatively, increased presence of TAMs, including M2-like TAMs specifically, is associated with an increased risk of vascular invasion in HNSCC.

### Increased CD68+ and CD163+ Density Is Associated With Higher Rate of Lymphatic Invasion

**Figure 8:**
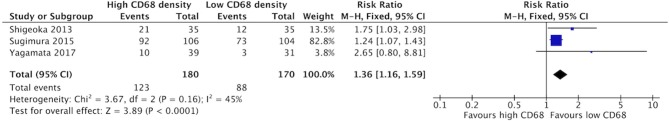
High CD68+ TAM density is associated with higher rates of lymphatic invasion.

**Figure 9:**
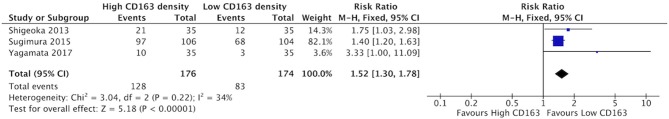
High CD163+ TAM density is associated with higher rates of lymphatic invasion.

Increased density of CD68+ in HNSCC samples was associated with higher risk of lymphatic invasion (Figure 8). Three studies contributed to a cumulative risk ratio of 1.36 (95%CI = 1.16–1.59, *p* < 0.0001). The average rate of lymphatic invasion was 57.5% in those samples with high CD68 density, and 38.1% in samples with low CD68 density. High density of CD163+ was associated with increased risk of lymphatic invasion (Figure 9). Three studies were identified, resulting in a risk ratio of 1.52 (95%CI = 1.30–1.78, *p* < 0.00001). The average rate of lymphatic invasion was 60.0% in samples with high CD163 density, and 36.1% in samples with low CD163 density. In summary, the presence of high levels of TAMs, including M2-like TAMs specifically, is associated with increased rates of lymphatic invasion.

### Increased CD68+ and CD163+ Density Is Not Associated With Poor Differentiation of Tumor

**Figure 10:**
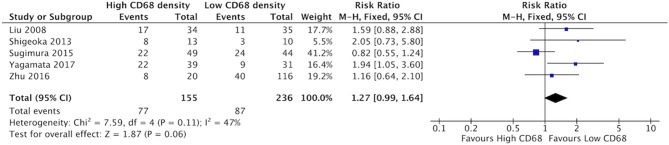
CD68+ TAM density is not associated with poor differentiation of tumor.

**Figure 11:**
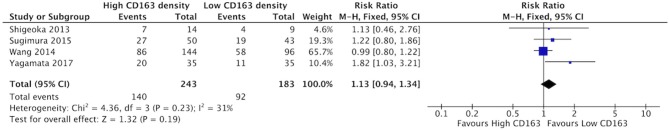
CD163+ TAM density is not associated with poor differentiation of tumor.

There was a trend suggesting high density of CD68+ TAMs may be associated with higher rates of poor differentiation of HNSCC, but this did not reach statistical significance (Figure 10). Five studies compared the correlation between these two factors, demonstrating a risk ratio of 1.27 (95%CI = 0.99–1.64, *p* = 0.06). The average rate of poor differentiation was 50.6% in samples with high CD68 density, and 35.9% in samples with low CD68 density. Presence of high levels of CD163+ density was also not associated with differentiation (*p* = 0.19) (Figure 11). Four studies were determined to examine this relationship. The resulting risk ratio was 1.13 (95%CI = 0.94–1.34, *p* = 0.19). The average rate of poor differentiation was 55.2% in those samples with high CD163 density, and 45.1% in samples with low CD163 density. While there is a trend approaching significance for CD163+ TAMs, neither CD163+ nor CD68+ staining correlated significantly with degree of tumor differentiation.

## Discussion

TAMs are a critical component of the TME in HNSCC and other cancers. Published studies evaluating TAMs have occasionally been contradictory, with high TAM levels both negatively and positively correlating with outcome (50, 51). Therefore this work aimed to evaluate the current literature on the prognostic relationship of this TME component. We conducted a meta-analysis on 12 studies that evaluated the association between TAMs and clinicopathologic factors of HNSCC. The analysis shows that an increase in total TAMs, particularly the M2-like subtype, was associated with a significant increase in risk for high T stage and nodal positivity. A significantly greater risk for lymphatic invasion and vascular invasion were also seen when TAMs were present in higher numbers in tumor tissue. In contrast, greater numbers of CD68+ as well as CD163+ TAMs were not found to be associated with poorly differentiated tumors.

The present study suggests a role of TAMs, notably those of the M2-like phenotype, in tumor growth, invasion, and metastasis. CD163-positive immunostaining is critical to identifying as well as activating this protumoral, M2-like subpopulation of TAMs (52, 53). Studies evaluating TAM RNA and protein products in HNSCC have identified that CD163-positive protumoral macrophages co-cultured with cancer cells release TGF-beta and epidermal growth factor (EGF) and upregulate ERK1/2, contributing to tumor growth and likely advanced T stage (54). TAMs also exhibit decreased expression of epithelial marker E-cadherin, and increased expression of mesenchymal markers Vimentin, Snail, and Slug, suggesting a role of TAMs in tumor cell epithelial-mesenchymal transition (EMT) (54, 55). The release of cytokines and chemokines in to the TME by M2-like macrophages, such as CCL16, CCL18, IL10, VEGF, Arginase 1, and YM1, may be responsible for accelerating cancer progression (56, 57). Antibodies blocking these secreted components are thus an attractive target for decreasing tumor cell replication, motility, and invasion.

It remains unclear whether the increased density of M2-like macrophages represents the majority of the total TAMs measured, and thus, accounts for similar results amongst the two groups. Two studies suggested that the population of total TAMs is skewed toward the M2-like phenotype. Yamagata et al. demonstrated nearly equivalent density of CD68 and CD163, with numbers in their samples ranging from 24 to 204/mm^2^ (median, 73mm^2^) vs. 22 to 156/mm^2^ (median, 73/mm^2^), respectively (51). Similarly, Fujii et al. noted that although the average number of CD68+ macrophages was slightly greater than the CD163+ macrophages, this difference was not statistically significant (2.72 cells/high-power field vs. 2.29 cells/high-power field) (42). The potential for M2-like macrophages to be responsible for the observed relation between TAMs and prognostic factors in head and neck squamous cell carcinoma is also corroborated in other cancer types (11, 58). Further studies should investigate whether CD163 indeed represents the majority of total TAMs in HNSCC tumoral tissue. If so, CD163 may act as a more specific marker for detection of tumor-promoting macrophages, and subsequently serve to assess, monitor, and potentially combat tumor growth and malignancy.

Given their importance in a variety of critical processes in the TME, interest has focused on TAMs as a therapeutic target. Strategies for targeting TAMs have shown promising results in preclinical trials, and several clinical trials are ongoing (34, 36, 59). Broadly, current TAM therapeutic strategies belong to one of four categories: (1) Depleting total TAM count; (2) Reducing recruitment of TAMs to primary tumor site; (3) Reprogramming M2-like macrophages to the tumoricidal M1-like phenotype; and (4) Limiting activation of TAMs. Trabectedin, which reduces total TAM number, was associated with reduced angiogenesis in both mouse tumor models and human sarcoma specimens (60). Metabolism agents with anti-tumor effects, such as metformin, may decrease M2-like abundance and polarization (61). Inhibition of the interaction between CD47, a marker of self upregulated by tumor cells in the TME, and SIRPα, a surface molecule on TAMs, enhances phagocytosis and decreases the M2/M1 ratio (62). Humanized anti-CD40 antibodies have been shown to re-educate M2-like TAMs to become M1-like, and induce tumor regression and improve survival in pancreatic cancer mouse models and surgically incurable patients (63). Inhibitors of the colony-stimulating factor 1 and its associated receptor (CSF1/CSF1R), which serve to generate monocyte progenitors and differentiate TAMs, have demonstrated benefit in skewing TAM population from protumoral to anti-tumoral predominance. Blockade of CSF1R in pancreatic ductal adenocarcinoma mouse models was shown to increase the activity of anti-tumor T cells, as well as improve response to PD-L1 checkpoint immunotherapy (64). Tumor cell-based activation of the Wnt/Beta-catenin pathway in TAMs is a newly identified and critical component of M2 polarization in cancer, particularly hepatic tumors (65). Disruption of Wnt pathway components with small-molecule inhibitors may be beneficial in diminishing numbers of M2 macrophages. Evidence suggests that TAM-associated treatment in conjunction with existing chemotherapy or radiation may provide the greatest benefit (65). Radiation induces DNA and cellular damage, leading to macrophage recruitment and subsequent promotion of tumor progression (66). Limiting the reactive infiltration of TAMs may promote improved responses to radiotherapy. Additionally, macrophage recruitment is often observed in tumors resistant to anti-angiogenic therapy, suggesting that TAMs may be related to drug resistance (67). Quantifying TAMs may thus be useful for prognostic stratification, as well as guidance for post-surgical therapy.

### Limitations

While this meta-analysis showed significant relationships between both total TAMs and M2 TAMs and multiple clinicopathologic indicators of poor prognosis, our analysis was limited in several ways. First, studies that do not demonstrate statistical significance are less likely to be published, which may skew our trends toward significance. Furthermore, multiple studies identified in the initial literature search provided data that was not amenable to statistical analysis, reporting TAM markers by percentage of total TAMs or simply displaying calculated *p-*values. This study reveals a correlation between TAMs and poor prognostic markers; however, a causative relationship would require further study. Mechanistic studies may help to define the timeline of TAM recruitment and thus, clarify their role in tumor progression.

In addition, there was not uniformity in the treatment that patients received prior to analysis. Some studies included patients who received chemotherapy prior to surgery, and treatment with radiotherapy and chemotherapy is known to alter the cellular composition of the TME. Moreover, timing of the sample collection may contribute to the populations of TAMs in majority, and thus, the levels of markers identified. It has been suggested that M1 macrophages are present in highest numbers when tumors first develop, and a gradual shift to M2 phenotype occurs as the tumor grows and spreads. A significant increase in M2 polarization occurs even during the periods between biopsy and tumor resection (67). While often considered dichotomous for the purpose of analysis, it should be reiterated that TAMs exist on a dynamic spectrum with some cells staining positive for both M1 and M2 markers (5). Subtypes within the M2 phenotype have been described, each elicited by different cytokine signals (68). Whether macrophages exist in these two polarized states is a matter of great debate and many authors feel that there is instead a wide spectrum of phenotypes of macrophages (69).

## Conclusions

Despite growing interest in TAMs, much is still unknown regarding their development, regulation, and diversity. As macrophage-focused treatments begin to gain clinical relevance, it will become necessary to elucidate the best mechanisms for utilizing TAMs in diagnosis, prognosis, and management. Within each of the categories of treatment described, multiple mechanisms to alter TAMs have been proposed and are actively under investigation. Responses in specific HNSCC subtypes will require further characterization and description, including investigation into which combination therapies provide the optimal tumor response.

Taken together, this meta-analysis lends weight to the growing interest in TAMs as a prognostic indicator in cancer. The literature demonstrates that elevated levels of TAMs, particularly of the M2 subtype, are related to poor clinicopathologic findings in HNSCC. Novel therapeutics targeting TAMs are an exciting avenue for targeted therapeutic strategies for HNSCC, and warrant further investigation.

## Author Contributions

JC designed the project. AKu, AKn, and BS performed the literature review, selected papers, collected the data, utilized RevMan to analyze the findings and generate forest plots, and wrote the manuscript. AKu, AKn, BS, UM-O, LH, NP, UR, AL, DC, JJ, and JC read, edited, and approved the final manuscript.

### Conflict of Interest Statement

The authors declare that the research was conducted in the absence of any commercial or financial relationships that could be construed as a potential conflict of interest.
